# Episcleritis

**Published:** 1887-08

**Authors:** R. O. Cotter

**Affiliations:** Macon, Ga.


### EPISCLERITIS.

---

BY R. O. COTTER, M. D., MACON, GA.

---

This disease, when chronic, will usually tax alike the skill and therapeutic resources of the physician, together with the patience and confidence of the patient. Stellwag, in his voluminous textbook on the eye, gravely dismisses the subject of episcleritis without a line regarding its treatment. I have had four cases within the past twelve months. Three of them were chronic, and, greatly to my gratification, if not surprise, they have all terminated in recovery. The disease, when chronic, is usually a most obstinate one, and yet is not such a very dangerous one; that is, it may only endanger the sight to the extent of the opacities left in the cornea. In rare cases the sclera may be so thinned at points as to result in ectosia or bulging at these atrophied places. It appears as a small, dusky, red, slight elevation over the sclerotic close to the insertion of one of the recti muscles, and usually the external one. There is usually more or less subconjunctival redness, and the tissues are of a dark, rusty or purplish hue. Sometimes the conjunctiva is not involved, and the vascularity and swelling of the tissues beneath the conjunctiva can be clearly seen. There may or may not be considerable photophobia. Usually there is some, and frequently there is considerable ciliary neuralgia and some lachrymation. The disease is liable, at the outset, to be mistaken for a more common one, herpetic or phlyctenular conjunctivitis; but in episcleritis the dusky little nodule does not break down and ulcerate.

The acute form of the disease is rarer than the chronic. In the chronic form the cornea finally becomes cloudy at the periphery. It appears to be an infiltration of lymph, and also seems that the opacity is brought about by the cutting off of its nutrition on account of the infiltration around the corneal periphery. Hence deep and permanent opacities may be the result.

As to the treatment, the text-books are very scanty in offering any decided treatment of this disease. After trying various plans I find the following to be of most service:

These cases will, in the majority of instances, be of a scrofulous diathesis, though in the acute form I think they are quite frequently due to simply a run-down condition of the system from almost any cause. The three cases of the chronic form referred to were of a scrofulous, or one of them was of a phthisical diathesis. These patients were put upon cod liver oil, iod. potass. 10 gr. three times daily. Good, nourishing diet; abstinence from all trashy food or sweet-meats. All the out-door exercise possible was also advised. A 2 gr. to the ounce solution of atropia was also used, both for its general sedative effect and specially hoped-for influence in aiding to remove the corneal opacities. Blisters were frequently applied to the temples, and with good effect, especially upon the ciliary neuralgia. In one case the diseases existed in only one eye, by no means an uncommon condition.

All these three cases went for months with occasional perceptible improvement, and as frequently they relapsed. I finally increased the dose of iod. potass. to 20 gr. with, I am confident, better results. A local application of three parts of oil of turpentine to five parts of olive oil was used two or three times daily in the eye to aid in the removal of the opacities.

The atropia was not kept up during the whole course of treatment. In the absence of iritis and corneal ulceration, I think the possibility of producing glaucoma should deter any conservative practitioner from using atropia for a considerable length of time. In the three chronic cases I alluded to, the corneal opacities have about cleared up and the vision of each patient is normal. I should also mention that we very frequently see syphilitic episcleritis. This yields readily to anti-syphilitic treatment.

